# Effects of pitavastatin on atherosclerotic-associated inflammatory biomarkers in people living with HIV with dyslipidemia and receiving ritonavir-boosted atazanavir: a randomized, double-blind, crossover study

**DOI:** 10.1186/s12981-023-00506-2

**Published:** 2023-02-27

**Authors:** Sirawat Srichatrapimuk, Artit Wongsa, Somnuek Sungkanuparph, Sasisopin Kiertiburanakul, Boonrat Tassaneetrithep, Angsana Phuphuakrat

**Affiliations:** 1 Chakri Naruebodindra Medical Institute, Faculty of Medicine Ramathibodi Hospital, Mahidol University, Samut Prakan, Thailand; 2 Center of Research Excellence in Immunoregulation, Faculty of Medicine Siriraj Hospital, Mahidol University, Bangkok, Thailand; 3 Division of Infectious Diseases, Department of Medicine, Faculty of Medicine Ramathibodi Hospital, Mahidol University, 270 Rama VI Road, Ratchathewi, Bangkok, 10400 Thailand

**Keywords:** Atherosclerosis, Dyslipidemia, HIV infection, Inflammatory biomarker, Pitavastatin

## Abstract

**Background:**

Chronic inflammation has been described in people living with HIV (PLHIV) receiving antiretroviral therapy (ART) despite viral suppression. Inflammation associated non-communicable diseases, including atherosclerosis, are becoming recognized complication of HIV infection. We studied the effect of pitavastatin on atherosclerotic-associated inflammatory biomarkers in PLHIV receiving ART.

**Methods:**

A randomized, double-blind, crossover study was conducted in HIV-infected persons with dyslipidemia and receiving atazanavir/ritonavir (ATV/r) to evaluate the effect of 2 mg/day pitavastatin treatment versus placebo. High-sensitivity CRP (hs-CRP), cytokines, and cellular markers in PLHIV receiving 12 weeks of pitavastatin or placebo were investigated.

**Results:**

A total of 24 HIV-infected individuals with a median (interquartile range) age of 46 (41–54) years were recruited, and the median CD4 T cell count was 662 (559-827) cells/mm^3^. The median duration of ATV/r use was 36 (24–48) months. Significant change in levels of basic fibroblast growth factor (FGF) between pitavastatin treatment and placebo at week 12 from baseline was observed (27.1 vs. 20.5 pg/mL; p=0.023). However, there were no significant changes from baseline of hs-CRP and other plasma cytokine levels at week 12 of pitavastatin or placebo. Regarding cellular markers, percentages of HLA-DR^+^CD38^-^CD4^+^ T cells and PD1^+^CD4^+^ T cells significantly decreased from baseline in PLHIV receiving pitavastatin for 12 weeks, as compared to placebo (− 0.27 vs. 0.02%; p=0.049 and − 0.23 vs. 0.23%; p=0.022, respectively).

**Conclusions:**

Pitavastatin treatment increases basic FGF levels, and lowers HLA-DR^+^CD38^-^CD4^+^ T cells, and PD1^+^CD4^+^ T cells. Further study on the effects of pitavastatin on preventing cardiovascular diseases in PLHIV should be pursued.

## Introduction

The use of antiretroviral therapy (ART) in people living with human immunodeficiency virus (PLHIV) has evidently reduced opportunistic infection-related morbidity and mortality [1]. Despite undetectable plasma viral load, immune activation and inflammation persist in PLHIV receiving ART [2]. These are associated with an increased risk of non-AIDS related morbidities and mortality. In the era of ART, the incidence of cardiovascular disease (CVD) was raised and become one of the leading causes of death in PLHIV [3]. PLHIV have an increased risk for CVD approximately 1.5-2 times compared to HIV-uninfected persons [4].

Protease inhibitors are currently recommended as alternative agents for first-, second-, and third-line antiretroviral regimens [5]. Among protease inhibitors, ritonavir-boosted atazanavir is not associated with increased risk of cardiovascular disease [6, 7], in contrast to other agents [7, 8]. Newer classes of antiretroviral agents, for example integrase strand transfer inhibitors, have emerged and are preferred in the first-line antiretroviral regimens [5]. However, long-term cardiovascular disease risk associated with these agents have yet to be confirmed in large-scale studies [9]. Although ritonavir-boosted atazanavir is not associated with the increased risk of cardiovascular disease, ritonavir-boosted atazanavir has greater increases of cholesterol and triglyceride levels from baseline compared to unboosted atazanavir [10].

Statins possess pleiotropic anti-inflammatory activities in addition to lipid-lowering effects [11]. Many inflammatory markers were shown to be related with atherosclerosis and cardiovascular diseases, such as interleukin-6 (IL-6), D-dimer, and high-sensitivity C-reactive protein (hs-CRP) [12, 13]. Use of different statins has been shown to be associated with reduced biomarkers of inflammation/immune activation and endothelial dysfunction, although there were some discrepant results [11, 14]. Pitavastatin has less drug-drug interactions compared with other lipid-lowering agents [15]; therefore the drug benefits for the use in PLHIV receiving ART that has drug-drug interaction with other statins. Limited data are available regarding the anti-inflammatory effect of pitavastatin in HIV-infected individuals. We studied the effect of pitavastatin use in virologically-suppressed HIV-infected patients on atherosclerotic-associated inflammatory biomarkers.

## Materials and methods

### Study design and participants

**Fig. 1:**
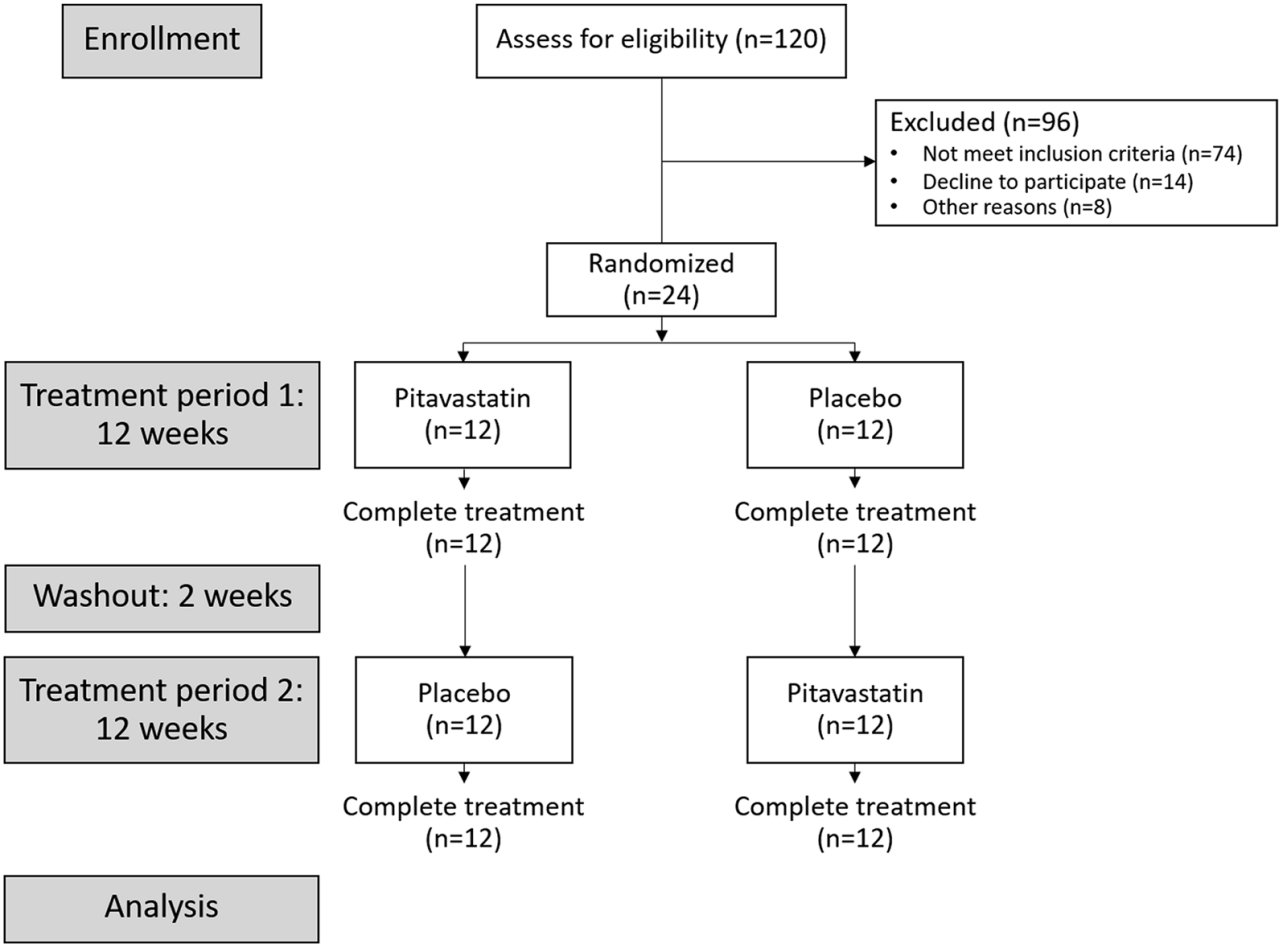
Flowchart of subjects in the randomized crossover trial

This study is a substudy of a randomized, double-blind, crossover study of 24 HIV-infected dyslipidemic patients receiving ritonavir boosted atazanavir (ATV/r) that was conducted to study safety and efficacy of pitavastatin for treatment of dyslipidemia (ClinicalTrials.gov NCT02442700) [16]. Breifly, participants were assigned to receive 2 mg/day of pitavastatin or placebo for 12 weeks, followed by 2 weeks of washout period, and then 12 weeks of the other treatment arm (Fig. 1). At the enrollment and at the end of 12 weeks of each treatment arm, blood was collected for inflammatory marker study. Estimated 10-year cardiovascular disease risk was calculated by Thai CV risk score, a tool developed to predict a 10 year cardiovascular risk using data from Thai people, as recommended by Thai guidelines [17, 18] (Application available on App store: https://apps.apple.com/id/app/thai-cv-risk-calculator/id1564700992).

The study protocol was reviewed and approved by the Ethical Clearance Committee on Human Right Related to Research Involving Human Subjects of the Faculty of Medicine Ramathibodi Hospital, Mahidol University (MURA2014/18).

### Immunophenotyping

Peripheral blood mononuclear cells (PBMCs) from study donors were isolated by gradient centrifugation using Histopaque^®^ (Sigma-Aldrich, Saint Louis, MO, US) as per manufacturer’s recommendation. Each sample was resuspended with 10% dimethyl sulfoxide (Sigma-Aldrich, MO, US) and 90% fetal bovine serum (Gibco, Carlsbad, CA, US) and frozen before use. After PBMC collection had been complete, samples were thawed in complete medium (RPMI 1640 medium) (Gibco, Carlsbad, CA, US) supplemented with 10% fetal bovine serum (Gibco, Carlsbad, CA, US). PBMCs were stained with LIVE/DEAD™ Fixable Aqua Dead Cell Stain (Invitrogen, Carlsbad, CA, US). Then, PBMCs were stained with fluorescence conjugated antibodies specific to protein surface markers, including PE/Cy5 anti-human CD25 (1:80), PE/Cy7 anti-human CD38 (1:80), APC/Cy7 anti-human CD4 (1:80), BV650 anti-human CD127 (1:80), Pacific blue anti-human CD3 (1:320), APC anti-human CD142 (1:160), FITC anti-human CD14 (1:40), PE-Texas Red anti-human CD16 (1:80), Alexa Fluor 700 anti-human CD8 (1:160) (BioLegend, San Diego, CA, US), and PE anti-human programmed cell death-protein 1 (PD-1) (1:40) (BD Pharmingen, San Jose, CA, USA), and eFluor 605NC anti human HLA-DR (1:40) (eBioscience, San Diego, CA, US). All samples were examined via BD LSRFortessa (BD Bioscience, San Jose, CA, USA). Data were analyzed using FlowJo software version 10.7.0 (Tree Star, Ashland, OR, US). Each cell subpopulation was estimated in percentage from total PBMCs without dead cells, debris, and doublet.

### Cytokine and chemokine levels

Levels of cytokines and chemokines in plasma samples were measured in duplicate by multiplex bead array assays using a Bio-Plex Pro Human Cytokine 27-Plex Assay Kit (Bio-Rad, Hercules, CA, USA) according to the manufacturer's instructions. The concentrations of interleukin (IL)-1β, IL-1ra, IL-2, IL-4, IL-5, IL-6, IL-7, IL-8, IL-9, IL-10, IL-12 (p70), IL-13, IL-15, IL-17, interferon-γ (IFN-γ)**,** tumor necrosis factor-α, eotaxin, basic fibroblast growth factor (FGF), granulocyte colony-stimulating factor (G-CSF), granulocyte-macrophage (GM)-CSF, IFN-γ-induced protein 10 (IP-10), monocyte chemoattractant protein-1 (MCP-1), macrophage inflammatory protein (MIP)-1α, MIP-1β, platelet derived growth factor BB (PDGF-BB), regulated upon activation, normal T cell expressed and secreted (RANTES), and vascular endothelial growth factor (VEGF) were analyzed in Bio-Plex Manager software (Bio-Rad, Hercules, CA, USA). In addition, hs-CRP levels were determined by a particle enhanced immunoturbidimetry assay (Rapid Labs, Colchester, Essex, UK) as per manufacturer’s recommendation.

### Statistical analysis

Median, interquartile range (IQR), and frequency were used to describe patients’ characteristics. Changes of levels of cytokines and proportions of immune cell subsets from baseline were compared by Wilcoxon signed ranks test. A *p*-value of <0.05 was considered statistically significant. Statistical analyses were performed using Stata statistical software version 17.0 (StataCorp, College Station, TX, US), and GraphPad Prism version 9.5.0 (GraphPad Software, San Diego, CA, US).

## Results

### Patient characteristics

Baseline characteristics of the 24 participants were shown in Table 1 [16]. Breifly, the median (IQR) age was 46 (41–54) years, and 14 participants (58.3%) were men. Of the participants, 95.8% had undetectable plasma HIV viral load, and the median CD4 T cell count was 662 (559–827) cells/mm^3^. The median duration of ATV/r use was 36 (24–48) months. The median 10 year estimated cardiovascular diseases risk was 2.7 (1.9–5.4) percent.

**Table 1:** Baseline characteristics of the participants

Characteristics	n=24
Age, years (median, IQR)	45.5 (41.0–54.0)
Male, %	58.3
CD4+ T cell count, cells/mm^3^ (median, IQR)	661.5 (559.3–827.0)
Undetectable HIV viral load, %	95.8
Duration of ATV/r use, months (median, IQR)	36 (24–48)
Comorbidities, %	
None	50.0
Dyslipidemia	25.0
Chronic HBV or HCV	16.7
Cardiovascular risk factors*, %	
<2	74.0
≥2	25.0
10-year CVD risk, % (median, IQR)	2.7 (1.9–5.4)
Baseline lipid profiles, mg/dL (median, IQR)	
Total cholesterol	242.5 (215.0–264.0)
LDL-cholesterol	144 (127.0–160.0)
HDL-cholesterol	41.0 (34.0–46.0)
Triglyceride	190.0 (145.0–355.0)

^*^Current smoking, systolic blood pressure ≥140 mmHg or on antihypertensive drugs, HDL-cholesterol <40 mg/dL, premature coronary heart disease in first-degree relative (age <55 years in male and <65 years in female), and age >45 years in male or >55 years in female

*ATV/r* atazanavir/ritonavir, *CVD* cardiovascular risk, *HBV* hepatitis B virus, *HCV* hepatitis C virus, *HDL* high-density lipoprotein, *IQR* interquartile range, *LDL* low-density lipoprotein

### hs-CRP, chemokine and cytokine levels

**Fig. 2:**
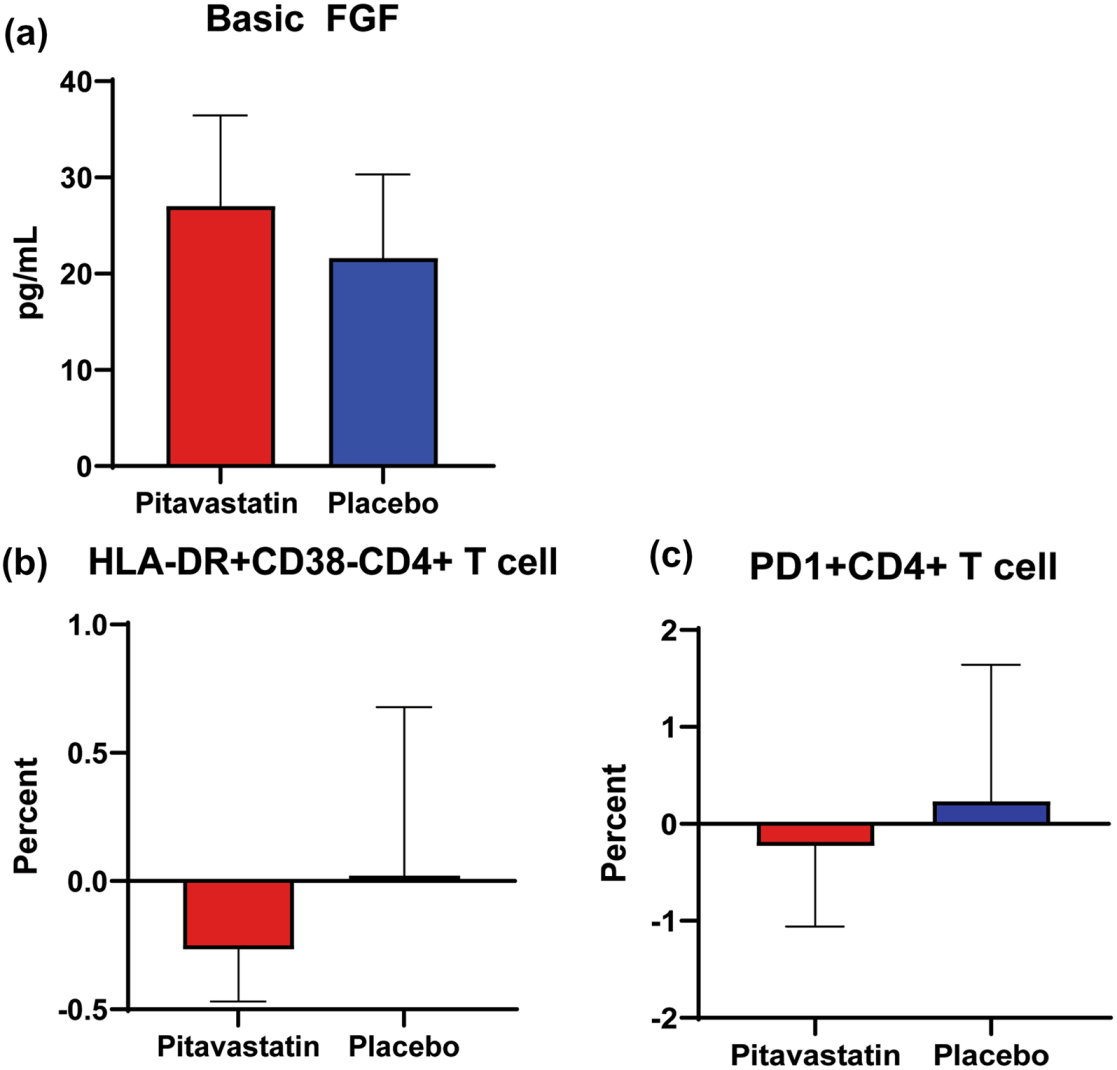
Inflammatory markers that revealed significant difference of changes from baseline to week 12 of pitavastatin vs. placebo in PLHIV receiving ritonavir-boosted atazanavir; (**A**) basic FGF, (**B**) HLA-DR^+^CD38^-^CD4^+^ T cells, (**C**) PD-1^+^CD4^+^ T cells. Y axis shows median change from the baseline

Pitavastatin did not have a significant effect on plasma levels of hs-CRP. A median change in levels of basic FGF at week 12 of pitavastatin treatment from baseline were significantly different compared to that after receiving placebo (27.1 vs. 20.5 pg/mL; p=0.023) (Fig. 2A). Change in level of IL-15 from baseline were increased after receiving placebo, but the difference of change after receiving pitavastatin vs. placebo did not reach statistically significance. Changes of levels of other cytokines from baseline were not different at week 12 after receiving pitavastatin vs. placebo (Table 2).

**Table 2:** Baseline and changes of cytokine levels from baseline to week 12 of pitavastatin treatment vs. placebo

Cytokines	Baseline	Change from baseline after pitavastatin	Change from baseline after placebo	*P*-value
IL-1β	2.01 (1.71 –2.42)	1.28 (0.39–2.87)	0.99 (0.30 –1.88)	0.820
IL-1ra	153.80 (132.90–180.80)	37.10 (− 13.40–81.50)	33.10 (8.44 –105.00)	0.865
IL-2	9.51 (6.32–11.16)	2.16 (0.49–5.54)	1.74 (0.65–3.54)	0.872
IL-4	3.89 (3.46–4.70)	1.82 (1.21–2.37)	1.35 (1.08 –2.20)	0.261
IL-6	7.66 (6.55 –9.50)	6.73 (1.00–33.3)	9.29 (0.09 –26.40)	0.966
IL-7	20.59 (11.55–34.30)	− 0.32 (− 15.10–4.72)	1.38 (− 12.70–11.70)	0.560
IL-8	23.87 (20.81–29.57)	57.00 (18.50–89.70)	53.6 (5.03–171.00)	0.551
IL-9	66.40 (53.63–76.46)	42.1 (21.10–53.90)	34.20 (14.80–66.00)	0.922
IL-10	14.38 (3.38–17.04)	2.19 (0.00–10.30)	2.36 (0.00 –10.50)	0.958
IL-12	19.24 (13.43–29.46)	9.39 (1.16–14.80)	8.94 (4.81–15.90)	0.601
IL-13	1.60 (1.16 –2.18)	1.23 (0.01–2.26)	1.06 (0.00–1.79)	0.768
IL-15	14.23 (11.49–19.67)	0.00 (− 4.03–1.57)	1.19 (− 2.34–3.14)	0.086
IL-17	211.20 (167.1 – 244.5)	173.00 (85.10–219.00)	129.00 (68.8–199.00)	0.747
Eotaxin	90.88 (70.86 – 110.20)	5.97 (− 6.60–17.70)	8.76 (− 9.80 –17.90)	0.790
Basic FGF	85.25 (75.04–98.90)	27.10 (18.30–36.60)	20.50 (11.60–29.00)	0.023
G-CSF	70.84 (59.80–76.97)	0.79 (− 11.40–10.80)	4.90 (− 3.33 –8.48)	0.622
GM-CSF	57.01 (48.31–66.63)	8.67 (0.24–14.40)	3.52 (0.00–13.00)	0.223
IFN-γ	14.40 (14.40–74.01)	0.00 (− 2.70–12.00)	0.00 (0.00–34.30)	0.269
IP-10	613.40 (466.00–921.90)	133.00 (17.30–201.00)	103.00 (20.70–158.00)	0.870
MCP-1	121.70 (100.80–160.50)	− 18.00 (− 49.10 – 0.62)	− 29.8 (− 43.80–−7.81)	0.520
MIP-1α	3.64 (1.00 –4.42)	6.64 (3.80–14.80)	6.21 (3.56–20.90)	0.716
MIP-1β	443.90 (368.50–482.90)	610.00 (188.00 –1618.00)	762.00 (87.80–1702.00)	0.368
PDGF-BB	409.70 (319.40–521.90)	359.00 (240.00–443.00)	305.00 (143.00–423.00)	0.601
RANTES	7240.00 (5799.00–9174.00)	− 2626.00 (− 5815.00–−506.00)	−1226.00 (− 3419–236.00)	0.107
TNF-α	52.70 (43.67–60.75)	25.10 (6.94–56.60)	21.90 (7.85-37.20)	0.475
VEGF	103.20 (81.34–164.90)	22.10 (− 12.10–47.90)	23.20 (0.07 –54.70)	0.331
hs-CRP	1.19 (1.00 –1.86)	0.12 (− 0.21–0.67)	0.17 (− 0.27 –0.78)	0.596

Values are shown as median (interquartile range). Units of cytokines are pg/mL; unit of hs-CRP is mg/L

*FGF* fibroblast growth factor, *G-CSF* granulocyte colony-stimulating factor, *GM-CSF* granulocyte-macrophage colony-stimulating factor, *hs-CRP* high-sensitivity C-reactive protein, *IFN* interferon, *IL* interleukin, *IP-10* interferon-γ-induced protein 10, *MCP-1* monocyte chemoattractant protein-1, *MIP* macrophage inflammatory protein, *PDGF-BB* platelet-derived growth factor BB, *RANTES* regulated upon activation, normal T cell expressed and secreted, *TNF* tumor necrosis factor, *VEGF* vascular endothelial growth factor

### Cellular immune profiles

For the study of cellular biomarkers, pitavastatin treatment did not have a significant effect on changes in the proportion of T cells, CD4^+^ T cells, CD8^+^ T cells, and monocytes (Table 3). However, pitavastatin treatment resulted in significant different changes in the proportion of HLA-DR^+^CD38-CD4^+^ T cells and PD-1^+^CD4^+^ T cells from baseline to week 12 of pitavastatin vs. placebo (− 0.27 vs. 0.02%; p=0.049, and − 0.23 vs. 0.23%; p=0.022, respectively) (Fig.2B, C).

**Table 3:** Baseline and changes in the proportions of immune cell subsets from baseline to week 12 after pitavastatin treatment vs. placebo baseline

Immune cell subsets (%)	Baseline	Change from baseline after pitavastatin	Change from baseline after placebo	*P*-value
Lymphocytes	55.50 (50.40–60.89)	− 2.10 (− 8.67–6.60)	−0.30 (− 7.50–16.00)	0.241
T cells	38.70 (19.91–45.93)	0.96 (− 9.07 –8.37)	0.12 (− 14.80 –6.83)	0.101
CD4^+^ T cells	19.72 (9.20–22.01)	− 0.78 (− 4.90–6.34)	1.66 (− 2.98–9.32)	0.134
HLA-DR^+^CD38^+^CD4^+^ T cells	0.84 (0.46–1.64)	− 0.07 (− 0.47–0.39)	0.04 (− 0.24–0.88)	0.205
HLA-DR^+^CD38^-^ CD4^+^ T cells	1.37 (0.86–1.95)	− 0.27 (− 0.47–0.24)	0.02 (− 0.35–0.68)	0.049
HLA-DR^-^CD38^+^CD4^+^ T cells	8.28 (3.90–11.59)	− 0.35 (− 3.06–2.21)	0.90 (− 1.24–5.45)	0.073
HLA-DR^-^CD38^-^CD4^+^ T cells	6.08 (1.75–7.97)	0.04 (− 1.12 – 2.65)	0.66 (− 0.91–2.96)	0.966
PD-1^+^CD4^+^ T cells	3.37 (1.07–4.27)	− 0.23 (− 1.06–0.79)	0.23 (− 0.92–1.64)	0.022
Regulatory T cells	0.76 (0.39–1.04)	0.09 (0.00–0.42)	0.23 (0.06 – 0.52)	0.097
CD8^+^ T cells	17.79 (8.51 – 21.32)	− 0.61 (− 4.02–3.47)	0.73 (− 3.96–5.20)	0.252
HLA-DR^+^ CD38^+^ CD8^+^ T cells	2.93 (1.64–3.48)	− 0.53 (− 1.08–0.59)	− 0.34 (− 1.26–0.83)	0.160
HLA-DR^+^CD38^-^CD8^+^ T cells	3.35 (2.38–5.01)	0.11 (− 0.92–1.59)	0.55 (− 0.50–1.76)	0.364
HLA-DR^-^CD38^+^CD8^+^ T cells	2.66 (1.26–5.74)	− 0.41 (− 1.00–0.16)	0.06 (− 0.72–1.33)	0.145
HLA-DR^-^ CD38^-^CD8^+^ T cells	5.07 (2.06–8.34)	0.60 (− 1.35–3.04)	− 0.12 (− 0.93–1.74)	0.703
PD-1^+^CD8^+^ T cells	2.88 (0.94 – 4.80)	− 0.17 (− 0.94 – 0.62)	− 0.01 (− 1.11–1.49)	0.426
Monocytes	3.46 (2.66–4.11)	1.07 (− 1.12 –1.64)	0.61 (− 0.76–1.92)	0.313
Classical monocytes	1.65 (0.90–2.71)	0.72 (− 0.60–1.81)	0.65 (− 0.40–2.18)	0.569
Intermediate monocytes	0.08 (0.04–0.12)	− 0.01 (− 0.05–0.03)	0.00 (− 0.03–0.05)	0.966
Nonclassical monocytes	0.31 (0.20–0.47)	0.03 (− 0.15 –0.)	0.02 (− 0.11–0.17)	0.326
Patrolling monocytes	0.02 (0.01–0.03)	0.00 (− 0.01 –0.02)	0.00 (− 0.01–0.02)	0.490

Values are shown as median (interquartile range)

## Discussion

We investigated the effects of pitavastatin on atherosclerotic-associated inflammatory biomarkers in PLHIV who had dyslipidemia and receiving ritonavir-boosted atazanavir. Although pitavastatin significantly lowered serum total cholesterol and LDL-cholesterol [16], there were no significantly different change of hs-CRP from baseline to week 12 of pitavastatin vs. placebo. However, pitavastatin treatment resulted in different changes of basic FGF, HLA-DR^+^CD38^-^CD4^+^ T cells, and PD1^+^CD4^+^ T cells from baseline to week 12 of pitavastatin vs. placebo.

The INTREPID study compared the effects of pitavastatin (4 mg daily) versus pravastatin (40 mg daily) on markers of immune activation and arterial inflammation in PLHIV and revealed greater reduction in soluble CD14, oxidized LDL, and lipoprotein-associated phospholipase 2, which are markers of immune activation and arterial inflammation, in the pitavastatin arm at week 52 [19]. We did not investigate these markers. However, similar to findings in our study, their study demonstrated no change in the levels of IL-6 and hs-CRP after one year of pitavastatin treatment [19, 20]. Our work revealed statistically difference in change of basic FGF levels between pitavastatin treatment and placebo at week 12. Basic FGF improves myocardial perfusion in animal model [21]. We also found an increase of IL-15 levels in the participants after receiving placebo, but the difference did not reach statistical significance. IL-15 is a pro-inflammatory cytokine that is up-regulated in atherosclerosis and myocardial infarction [22]. Further studies with larger sample size and/or increased dose of pitavastatin should be performed. However, the use of 4 mg/day of pitavastatin was associated with more muscle complaints compared to the use of 1 mg/day dose [23].

Our results showed significantly different changes in the proportions of T cell subsets of HLA-DR^+^CD38^-^CD4^+^ T cells and PD-1^+^ CD4^+^ T cells from baseline to 12 week of pitavastatin vs. placebo. The previous study demonstrated significant reductions in HLA-DR^+^CD4^+^, HLA-DR^+^CD8^+^, and HLA-DR^+^CD38^+^ T cells after receiving 8 weeks of high dose atorvastatin (80 mg daily) [24]. We also found reductions in HLA-DR^+^ in CD4^+^ T cells, but only the HLA-DR^+^CD38^-^CD4^+^ T cell subpopulation yielded statistically significant difference. A previous study showed that an increased HLA-DR^+^ T cells in hypercholesterolemic subjects as well as in patients with chronic stable angina and acute myocardial infection compared with controls [25]. HLA-DR is recognized as a marker of T cell activation. Therefore, pitavastatin treatment might lower T cell activation and hence reduce immune activation in PLHIV. PD-1 is one of the immunosuppressive costimulatory molecules, which mediates an inhibitory effect [26]. PD-1 is up-regulated on HIV-specific CD4^+^ T cells and its expression level correlated with plasma viremia and inversely with CD4^+^ T cell counts [27]. PD-1 is a marker of T cell exhaustion, and can be found during chronic infection [28]. T cells from human atherosclerotic plaques were found to express high levels of PD-1 [29]. Okoye and co-workers previously demonstrated the reduction of co-inhibitor receptor expression, including PD-1, after atorvastatin treatment [30]. This suggested the role of statin in restoring T cell function. Taken together, this study supported the decreased of some atherosclerotic-associated inflammatory markers after pitavastatin treatment.

The strength of our study included the double-blind, crossover study design which could minimize the biases. However, we accepted the limitations of the small sample size, and the short duration of the washout period. Some independent markers of cardiovascular disease-related mortality, i.e., sCD14, D-dimer, and fibrinogen, were not studied. Further, the short duration of follow-up did not allow for the detection of clinical outcome of cardiovascular events. A large prospective trial, for example, Randomized Trial to Prevent Vascular Events in HIV (REPRIEVE) [31], that determines the effect of long-term statin use in PLHIV on major adverse cardiovascular events would be helpful to answer this question.

## Conclusions

We demonstrated that pitavastatin treatment, in addition to lipid lowering effect, could lower some of the atherosclerotic-associated cellular markers, including the proportions of HLA-DR^+^CD38^-^CD4^+^ T cells, and PD-1^+^CD4^+^ T cells. We also found an increased levels of basic FGF, which has atherosclerotic-protective effects. Further study on the effects of pitavastatin on preventing cardiovascular diseases in PLHIV is warranted.

## Data Availability

The datasets used and/or analyzed during the current study are available from the corresponding author on reasonable request.

## Abbreviations

ART: Antiretroviral therapy
ATV/r: Atazanavir/ritonavir
CD: Cluster of differentiation
CVD: Cardiovascular disease
FGF: Fibroblast growth factor
G-CSF: Granulocyte-colony stimulating factor
GM-GSF: Granulocyte mocrophage-colony stimulating factor
HIV: Human immunodeficiency virus
hs-CRP: High-sensitivity C-reactive protein
IFN-γ: Interferon gamma
IL: Interleukin
IP-10: Interferon gamma-induced protein 10
IQR: Interquartile range
MCP-1: Monocyte chemoattractant protein-1
MIP-1α: Macrophage inflammatory protein-1 alpha
MIP-1β: Macrophage inflammatory protein-1 beta
PBMC: Peripheral blood mononuclear cell
PD-1: Programmed cell death-protein 1
PDGF-BB: Platelet derived growth factor BB
PLHIV: People living with HIV
RANTES: Regulated on activation, normal T cell expressed and secreted
TNF-α: Tumor necrosis factor alpha
VEGF: Vascular endothelial growth factor
